# American Medical Association at Boston

**Published:** 1906-06

**Authors:** 


A Monthly Review of Medicine and Surgery.

EDITOR:

WILLIAM WARREN POTTER, M. D.

All communications, whether of a literary or business nature, books for review and exchanges, should be addressed to the editor 284 FRANKLIN ST., BUFFALO, N. Y.

VOL. LXI. JUNE, 1906. NO. 11

### American Medical Association at Boston.

THE American Medical Association will meet in Boston for the third time in its history, June 5-8, 1906. The forthcoming meeting will be held under the administration of Dr. L. S. McMurtry, of Louisville, retiring president, and Dr. William J. Mayo, of Rochester, Minn., incoming president. The association was organised by a preliminary conference at New York in 1846, and a first regular session at Philadelphia in 1847, at which latter the organisation was perfected. The second meeting was held at Baltimore in 1848, and the third at Boston in 1849. The next meeting at Boston was held in 1865, just as the country was emerging from the civil war which had interrupted the annual sessions of the association. The second meeting at Boston, therefore, was the sixteenth annual session, while the coming meeting, which will be convened about the time this JOURNAL reaches our readers, will be the fifty-seventh annual meeting of the association.

At the meeting in 1865, the committee on medical education, acting on a suggestion presented by the Medical Society of the State of New York, recommended the passage of a law by each state establishing a board of medical examiners, whose function should be to license successful candidates to practise medicine, after the medical schools had issued diplomas to them. This recommendation by the Medical Society of the State of New York was made at the instance of the faculty of the University of Buffalo, as the following minute taken from the records of the college will attest:

UNIVERSITY OF BUFFALO, MEDICAL DEPARTMENT,
February 2, 1864.

On motion of Professor Charles A. Lee, seconded by Professor James P. White, it was

*Resolved,* That the Medical Society of the State of New York be requested to appoint a committee to consider the expediency of, and to report a plan for, the appointment of a state board of examiners for the degree of doctor of medicine, and to report at the next meeting of the society.

*Resolved,* That the same committee be instructed to bring the subject before the next meeting of the American Medical Association, and that the delegates of this society be instructed to urge the general adoption of the same plan in other states of the union. Carried unanimously.

Sanford Eastman, THOMAS F. ROCHESTER,
*Dean of the Faculty.* *Chairman.*

It is worthy of note that the American Medical Association referred this recommendation of the committee on medical education to the committee on publication *without discussion.* The attitude of the association toward this question has undergone a complete change of late, and its council on medical education now invites members of the examining boards to attend its annual conferences.

In relation to the fifty-seventh session about to convene at Boston there are many reasons why it should be the most important meeting, scientifically, socially, and numerically speaking, in its long and useful career. Boston is a center of education, culture, refinement and wealth; it is famous in its history, metropolitan in its proportions, and magnificent in its art collections; and it is easily accessible by rail and water. Moreover, this will be the first meeting in a quarter of a century to which the Medical Society of the State of New York has sent its official representatives, which fact in a measure will make the meeting distinctive. Arrangements are making through the appointed officers to make the gathering the largest and most representative medical convention ever held in the world. We trust these expectations will be realised in every respect.

---

A historical exhibition is announced to take place in London at an early day, under the direction of Henry S. Wellcome, Esq., who is an acknowledged authority on questions of medical history and an expert observer of scientific antiques. The exhibition will consist of rare and curious objects relating to medicine, chemistry, pharmacy and the allied sciences. It will be strictly professional and scientific in character and will not be opened to the general public. With the object of making this exhibition of large interest, Mr. Wellcome invites loans from persons in all parts of the world who feel an interest in the success of the undertaking and a willingness to contribute to that end.

The exhibition will contain portraits, paintings, drawings and all kinds of pictures; medals, medallions and coins of medical interest; rare and curious manuscripts, books, periodicals and other publications; letters, prescriptions, autographs and ancient diplomas; instruments, apparatus and chemical appliances; curiosities of surgery, anatomy, and pathology; placards, posters, and declarations concerning epidemic disease; and a multitudinous variety of other similar materials relating to medicine and the allied sciences. The extent of the work involved renders it impossible at present to fix a definite date, but announcement thereof will be duly made. Mr. Wellcome has issued an artistic illustrated announcement in which he invites loans for this most interesting, instructive and useful exhibit. All communications respecting the historical medical exhibition should be addressed to Mr. Henry S. Wellcome, Snow Hill Buildings, London, E. C. England.

---

It is a great satisfaction to the *Post-Graduate* to announce that the State Legislature declined to pass the osteopathic, or optometry, or any other bill allowing the practice of medicine in our state, without a fulfilment of the requirements of the medical law that went into effect in 1891, which places New York in the front rank as to high requirements for the practice of our art. This happy result is due to the labors of Dr. Arthur G. Root, Chairman of the Committee on Legislation of the State Society, with the coöperation of the profession and the medical press of the state. The osteopathic bill passed the Senate, but the Assembly advised by such men as Prentice, Agnew, and Francis, stood firmly for the protection of the people against quacks, and for a year more, and we hope for ever, we are safe. —*Post-Graduate,* May, 1906.

---

San Francisco is emerging from the first effects of its disaster with astonishing alacrity. Nevertheless, it will take a long time to restore her people to comfort or even to the bare necessities of life. Physicians in particular are hampered in their ministrations by reason of loss of their offices, equipment, instruments, books and all the usual appliances that are needful in everyday practice. Contributions in kind, as well as money, are urgently needed and we again invite the attention of the physicians of Buffalo, in particular, to this matter and hope they will respond to this appeal from their professional brethren who are the victims of such unfortunate and distressful conditions. Dr. Roswell Park, president of the Buffalo Red Cross should be communicated with by donors.

